# Transcriptome analysis of growth heterosis in pearl oyster *Pinctada fucata martensii*

**DOI:** 10.1002/2211-5463.12502

**Published:** 2018-10-12

**Authors:** Jingmiao Yang, Shaojie Luo, Junhui Li, Zhe Zheng, Xiaodong Du, Yuewen Deng

**Affiliations:** ^1^ Fisheries College Guangdong Ocean University Zhanjiang China; ^2^ Pearl Breeding and Processing Engineering Technology Research Center of Guangdong Province Zhanjiang China

**Keywords:** growth, heterosis, pearl oyster, *Pinctada fucata martensii*, transcriptome

## Abstract

Heterosis improves growth and survival of shellfish species. Although breeders have widely exploited heterosis, its underlying molecular mechanisms remain unclear. In this study, a 2 × 2 complete diallel cross was facilitated between two full‐sib families to produce two inbred families (A and D) and their reciprocal hybrid families (B and C) of pearl oyster *Pinctada fucata martensii*. Growth traits of the four families were compared at the adult stages. Transcriptome analysis was conducted on the four families using an Illumina sequencing platform. The results revealed that the growth traits of the four families significantly varied (*P *<* *0.05). The mid‐parent heterosis values of shell length, shell height, shell width, shell weight, and total weight were 12.9%, 14.9%, 18.2%, 17.2%, and 33.2%, respectively. The B‐ and C‐inbred (A and D) triads had 79 and 68 differentially expressed genes (DEGs), respectively, which were dominantly nonadditive, including overdominance, underdominance, and low‐parent dominance. Gene ontology term analysis showed that the DEGs in the B‐ and C‐inbred triads were enriched for metabolic process, cellular process cell part, binding, and catalytic activity. Kyoto Encyclopedia of Genes and Genomes pathway enrichment analysis indicated that the DEGs in the B‐ and C‐inbred triads were involved in focal adhesion, the P13K‐Akt signaling pathway, the mRNA surveillance pathway, and the focal adhesion pathway. The reliability of the sequencing data was confirmed by real‐time polymerase chain reaction analysis of six growth‐related genes. The findings of this study provide new insights into heterosis for growth traits and the design of genetic breeding programs for this species.

**Abbreviations**
CGRNP: cell growth‐regulating nucleolar protein
DEG: differentially expressed gene
IGFBP: insulin‐like growth factor‐binding protein
MYCBP2: MYC binding protein 2
rRM: ribosomal RNA methyltransferase
TBC: tubulin beta chain
ZP: zinc protein

Pearl oyster *Pinctada fucata martensii* is one of the most important components of molluscan mariculture in southern China, particularly in Guangdong, Guangxi, and Hainan provinces. This species is cultured to produce nucleated round pearls referred to as the ‘South China Sea Pearl’. However, pearl oyster cultures in these provinces have recently suffered from severe slow growth and mass mortalities. Breeding studies have been initiated to improve growth and resistance to diseases and increasingly deteriorating environments 1. These studies include mass selection 1, 2, 3 and population hybridization 4. Mass selection is an effective approach to improve the growth traits of *P. fucata martensii*. The fifth generation line selected for the faster growth rate of *P. fucata martensii* displayed a larger mean shell length and shell height than those of the control group 5. Hybridization is an alternative approach to improve the growth traits of *P. fucata martensii* by crossing geographically isolated populations and selected lines 4.

Heterosis or hybrid vigor refers to the phenomenon in which a hybrid offspring exhibits phenotypic superiority over its parents. It has been commonly utilized to improve the growth traits and survival of shellfish species 4, 6, 7, 8. Therefore, the genetic mechanisms of heterosis have gained considerable interest in genetics and breeding research. Although heterosis has been widely exploited by crop and animal breeders, its underlying molecular mechanisms remain poorly understood. Three classical hypotheses have been proposed to explain heterosis: dominance, overdominance, and epistasis. Dominance refers to the complementation by superior dominant alleles in a heterozygous hybrid. Overdominance ascribes allelic interactions at one or more loci for heterotic traits. Epistasis attributes heterosis to the epistatic interaction of beneficial nonallelic genes at two or more loci in hybrids 9.

With the rapid improvements and advancements in molecular biological methods, heterosis in crop and animals has been elucidated with molecular evidence. At present, several techniques are available for various aquatic species. These techniques include suppression subtractive hybridization, mRNA differential display techniques, and cDNA‐amplified fragment length polymorphisms. A high‐throughput sequencing of transcriptomes has been recently used to explore the molecular mechanism underlying heterosis 9. High‐throughput sequencing of the transcriptome analyses of *P*. *fucata martensii* identified numerous candidate genes involved in embryogenesis, growth traits, and biomerialization 10, 11. Recent works have reported on the genomes of *P*. *fucata martensii*, which served as basis for investigating of their important traits 11.

We have recently developed a pearl oyster breeding program to improve the growth performance of cultured stocks, and a large number of families have been developed. In a previous study, we conducted an experiment to compare several gene expression profiles between purebred and hybrid families by qRT‐PCR and found significantly different gene expression levels among the families 12. In this study, the whole transcriptomes of inbred and hybrid families were sequenced to analyze the functional differences among the four families and provide a basis for investigating the mechanisms of growth heterosis in the species.

## Materials and methods

### Experimental animals

A 2 × 2 complete diallel cross was made between two full‐sib families (M and N) to produce four families. The four families were named as follows: (a) A, an inbred family produced by sister–brother mating from family M; (b) B, a hybrid family with the female parent from family M and the male parent from family N; (c) C, a reciprocal hybrid family with the female parent from family N and the male parent from family M; and (d) D, an inbred family produced by sister–brother mating from family N.

The inbred and hybrid families were developed in March 2014. In the complete diallel cross, a female was mated to a full‐sib brother and an unrelated male to produce inbred and hybrid families. The larvae were reared following the techniques of Deng *et al*. 1. The larvae were reared in 1000‐L polyethylene tanks. The density was maintained at 1 individual·mL^−1^. Water temperature was 27.2 ± 1.2 °C, and salinity was 30.0 ± 0.5 ppt. Daily feeding consisted of *Isochrysis zhanjiangensis* from days 2 to 7 and a mixture of *I*. *zhanjiangensis* and *Chlorella* sp. from days 7 to 55. On Day 21, plastic plates were provided as substrate for metamorphosis. On Day 50, individuals as large as 3–5 mm were removed from the plates, transferred to net‐cages, and suspended in the sea of Liushagang, Zhanjiang. The shells were cleaned and placed in new nets at appropriate intervals.

### Growth measurement and sampling

A total of 100 individuals with 2 years of age were sampled from each family. Shell length, shell height, shell width, and shell weight of each individual were measured. Ten individuals were sampled from each family, and adductor muscle of each individual was dissected. Each sample was formed by pooling adductor muscles of equal size from two individuals within a family and then stored at −80 °C.

### Total RNA extraction, cDNA library construction

Total RNA of the sample was extracted with TRIzol reagent. Trichloromethane was used for protein denaturation and phenol extraction. Isopropanol was used to precipitate the nucleic acids, which were then dissolved in DEPC water. RNase‐free tips and tubes were used in RNA extraction at all times. The concentration of each sample as well as OD260/OD280 and OD260/OD230 was measured using SimpliNan. The total RNA, including RNA concentration, RIN value, 28S/18S, fragment length distribution, and cDNA library construction, was detected by an Agilent 2100 Bioanalyzer (Agilent RNA 6000 Nano Kit). Two samples were used for each family, and eight cDNA libraries were constructed.

### Data analysis of RNA‐seq

The project used internal software SOAPnuke to filter reads and remove reads with adaptors. Reads with unknown bases (N) were more than 5%. Low‐quality reads, which were defined as a read in which the percentage of the base is less than 15, were greater than 20%. Clean data were obtained in FASTQ format. The statistics included Q20, Q30, and the ratio of clean reads. The clean reads were used in the subsequent analysis. The clean reads were mapped to the reference genome using HISAT after the reads were filtered. The bam format date of the reads that contrasted with the genome sequences was obtained 11. The bam format date can be used by IGV. IGV can be compared with multiple samples to find the distribution of reads in exons, introns, UTRs, and intergenic regions.

### Differential expression analysis

Differentially expressed genes (DEGs) were detected by the DEGSeq method. [log_2_ Ratio (BP/SP)] > 1 and FDR≤0.001 were used as the threshold for a significant differential expression. On the basis of the DEGs, the classification and aggregation analysis of gene ontology (GO) function were performed and were divided into three major functions: cellular component, molecular function, and biological process. KEGG is a database resource from molecular level information to understand advanced functions and biological systems 13.

According to the method of Rapp *et al*. 14, the DEGs in a hybrid‐inbred (parent) triad were classified into five possible expression classes of differential expression, that is, the expression levels of genes equal to the mid‐parent (additivity), the high‐ or low‐parent (high‐ or low‐parent dominance), above the high‐parent (overdominance), or below the low‐parent (underdominance) 14. The mid‐parent value was calculated for two replicates and compared with the average hybrid expression for the replicates. A two‐tailed homoscedastic *t*‐test was performed. Genes with *P *>* *0.05 were considered to be additively expressed, whereas genes with *P *<* *0.05 were regarded as nonadditively expressed.

### Quantitative real‐time PCR

Gene expression profiles were validated by quantitative real‐time PCR (qRT‐PCR). The gene‐specific primers for qRT‐PCR were designed using primer 5.0 (Premier Biosoft International, Palo Alto, CA, USA) (Table 1). Ten individuals were used for each of the four families. The total RNA was extracted using TRIzol (Thermo Scientific TM). cDNA was synthesized using PrimeScript RT Reagent Kit (TaKaRa, Dalian, China) 15, 16. A 5 μL PCR mixture consisted of 2.5 μL of SYBR mixture (TaKaRa), 0.2 μL of cDNA, 0.2 μL of each PCR primer, and 2.1 μL of ddH_2_O. The PCR amplification procedure was 40 cycles of 50 °C for 2 min, 95 °C for 2 min, 95 °C for 15 s, 60 °C for 1 min, 95 °C for 15 s, 60 °C for 1 min, 95 °C for 30 s, and 60 °C for 15 s. The results were expressed by 2^−▵▵Ct^. Each sample consisted of three replicates. The relative expression of the genes was calculated by the 2^−▵▵Ct^ method, and the standard deviation was calculated among three replicates 17.

**Table 1:** Primer sequences for quantitative real‐time PCR

Gene ID	Forward Primer	Reverse Primer
PINCT_32260	GGAAATTACGGCCTCGCTGGTT	AGGCATCATCGTGGATTCTCTC
PINCT_23260	CCAAGTCCATTACGGAATCAGA	CGGCATCAGACTCACGGCAT
PINCT_18317	CCCAGACAAACGGAGTACAGAA	TTGTTGGCCTTCTTCCTTCTCT
PINCT_08355	ATGGAAGATTGGGATGAAAACC	CTCCGTTTTGACATTCCCAGA
PINCT_32812	CTGCTGTGATGCCAAGTCGAGT	TCGTCCGAAGTCCAGTCCCGTA
PINCT_09305	CTAGGTGGCGGTACTGGTGC	GTCAGATACTTTGGGGGAGGGT
*β*‐Actin	TGGTATGGGACAGAAGGAC	GACAATGCCGTGCTCAAT

### Statistical analysis

One‐way ANOVA followed by Tukey test was performed to compare the differences in the mean values of growth traits and gene expression of the four families. Significance level was set at *P *<* *0.05.

Mid‐parent heterosis (MPH) was calculated for shell length, shell height, shell width, shell weight, and total weight of the samples using the following equation: MPH(%) = [(*F*_1_−MP) × 100]/MP, where *F*_1_ is the mean shell length, shell height, shell width, shell weight, or total weight of two reciprocal hybrids; MP = (*P*_1_ + *P*_2_)/2, where *P*_1_ and *P*_2_ are the mean shell length, shell height, shell width, shell weight, and total weight of the two inbred families.

## Results

### Comparison of the growth traits of the four families

Significant differences were detected in the measured growth traits of the four families (*P *<* *0.05). These differences were observed between the inbred families, between the hybrid families, and between the inbred and hybrid families. Among the four families, hybrid C had the highest values for shell length, shell height, shell width, shell weight, and total weight. The MPH varied for the different growth traits with the values of shell length, shell height, shell width, shell weight, and total weight were 12.9%, 14.9%, 18.2%, 17.2%, and 33.2%, respectively (Table 2).

**Table 2:** Mean growth traits of inbred and hybrid families and mid‐parent heterosis calculated for growth traits

Groups	Shell length mm	Shell height mm	Shell width mm	Shell weight g	Total weight g
A	59.84 ± 3.2^b^	60.78 ± 2.7^b^	19.42 ± 1.4^ab^	16.46 ± 1.7^b^	23.38 ± 4.4^b^
B	64.56 ± 2.7^ab^	66.34 ± 3.9^a^	22.14 ± 1.5^a^	17.77 ± 1.9^a^	28.15 ± 3.6^a^
C	67.32 ± 3.5^a^	69.82 ± 4.4^a^	22.88 ± 1.7^a^	19.78 ± 2.3^a^	31.42 ± 4.7^a^
D	56.92 ± 2.3^c^	57.82 ± 3.4^b^	18.68 ± 1.2^b^	15.57 ± 1.2^b^	21.34 ± 3.3^b^
MPH%	12.9	14.9	18.2	17.2	33.2

Means with the same letter within a column are not significantly different (*P *>* *0.05). Values are means ± SD, *n* = 50.

### Transcriptome profiling of two hybrids and their parental families

After filtering low‐quality, joint contamination, and high‐content reads with an unknown base N, clean data were mapped to the genome using HISAT. The average ratios of alignment and unique mapping were 73.86% and 46.65% (Table 3). A total of 32 526 genes were detected, which included 28 156 known genes and 4430 predicted new genes. A total of 46 370 new transcripts were detected, of which 21 664 new alternative splice variants belonged to protein‐coding genes, 4430 belonged to new protein‐coding genes, and the remaining 20 276 belonged to long‐chain noncoding RNAs.

**Table 3:** Transcriptome mapping statistics

Sample	Total raw reads (Mb)	Total clean reads (Mb)	Total clean bases (Gb)	Clean reads Q20 (%)	Clean reads Q30 (%)	Clean reads ratio (%)	Total mapping ratio	Uniquely mapping ratio
A	80.94	66.51	6.65	96.10	87.27	82.20	73.47%	44.91%
B	79.69	66.06	6.61	96.22	87.62	82.90	74.54%	46.13%
C	78.45	66.90	6.69	96.90	89.23	85.47	74.72%	48.76%
D	74.71	66.28	6.63	97.69	91.40	88.72	72.72%	46.79%

### DEGs between inbred and hybrid families

We investigated the variation in DEGs by performing pairwise comparisons between the inbred families and their reciprocal hybrid families. Differences were observed in gene expression between the two inbred families (A and D). A total of 594 DEGs (346 were upregulated and 248 were downregulated) were identified between A and D. Meanwhile, 283 DEGs (47 upregulated and 236 downregulated) were identified between reciprocal hybrid families B and C (Table 4).

**Table 4:** Numbers of differentially expressed genes between inbred and hybrid families

Group	DEGs_total	DEGs_up	DEGs_down
A vs B	1305	862	443
A vs D	594	346	248
B vs C	283	47	236
B vs D	193	69	124
A vs C	316	204	112
C vs D	338	181	157
B vs A, D	79	42	37
C vs A, D	68	49	19

We also investigated the DEGs in the B‐ and C‐inbred triads. In the B‐inbred triad, 79 DEGs were identified, of which 42 DEGs were upregulated and 37 DEGs were downregulated. In the C‐inbred triad, 68 DEGs were identified, of which 49 DEGs were upregulated and 19 DEGs were downregulated (Table 4).

### DEGs expression model

**Figure 1:**
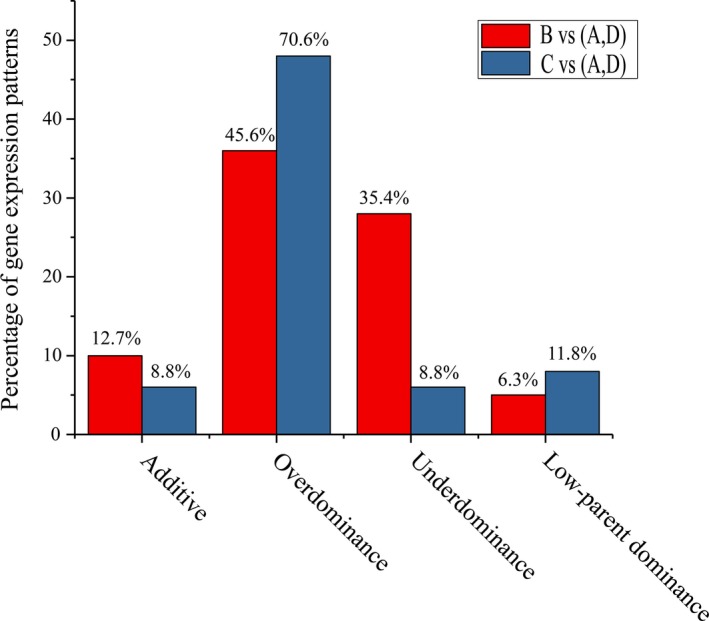
Distribution of DEGs expression patterns in the B‐ and C‐inbred triads.

We further investigated DEGs expression model in the B‐ and C‐inbred triads. In the hybrid B‐inbred triad, 69 (87.3%) genes exhibited nonadditive model and 10 (12.7%) genes exhibited additive model. Of 69 genes, 36 (45.6%), 28 (35.4%), and 5 (6.3%) genes showed overdominance, underdominance, and low‐parent dominance, respectively. In hybrid C‐inbred triad, 62 (91.2%) genes exhibited nonadditive model and six (8.8%) genes exhibited additive model. Of 62 genes, 48 (70.6%), 6 (8.8%), and 8 (11.8%) genes exhibited overdominance, underdominance, and low‐parent dominance, respectively (Fig. 1).

### Functional analysis of DEGs

The DEGs between the two inbred families (A and D) presented genetic differences in parental breeders. GO analysis showed that the DEGs between the two inbred families were enriched in the cellular and metabolic processes in biological processes; in the membrane and organelle of the cellular component; and in the binding and catalytic activities in molecular function. The enriched Kyoto Encyclopedia of Genes and Genomes (KEGG) pathway of the DEGs between the two inbred families included microRNAs in cancer, ECM–receptor interaction, PI3K‐Akt signaling pathway, and so on (Table 5).

**Table 5:** Enriched KEGG pathway of DEGs between the hybrid and inbred families

KEGG pathway	A vs B	B vs D	A vs C	C vs D	A vs D	B vs C
Calcium signaling pathway	24	5	3	12	14	6
Dilated cardiomyopathy	19	6	4	8	7	6
ECM–receptor interaction	48	5	8	10	35	6
Endocytosis	49	7	10	57	10	80
Epstein–Barr virus infection	33	3	13	9	10	9
Focal adhesion	70	8	17	64	32	83
HIF‐1 signaling pathway	31	5	9	55	8	78
Lysine degradation	30	3	10	4	8	4
MicroRNAs in cancer	53	7	4	12	41	9
mRNA surveillance pathway	56	5	12	83	7	108
NOD‐like receptor signaling pathway	27	3	13	51	16	55
Pathways in cancer	23	4	14	10	17	6
Phagosome	20	2	9	10	15	6
PI3K‐Akt signaling pathway	70	11	14	65	32	89
Purine metabolism	28	2	11	13	7	10
Rap1 signaling pathway	55	6	16	58	22	81
Ras signaling pathway	47	5	9	58	15	79
RNA transport	28	7	3	7	8	4
Spliceosome	24	3	4	4	10	4
Transcriptional misregulation in cancer	46	4	9	60	8	83

**Figure 2:**
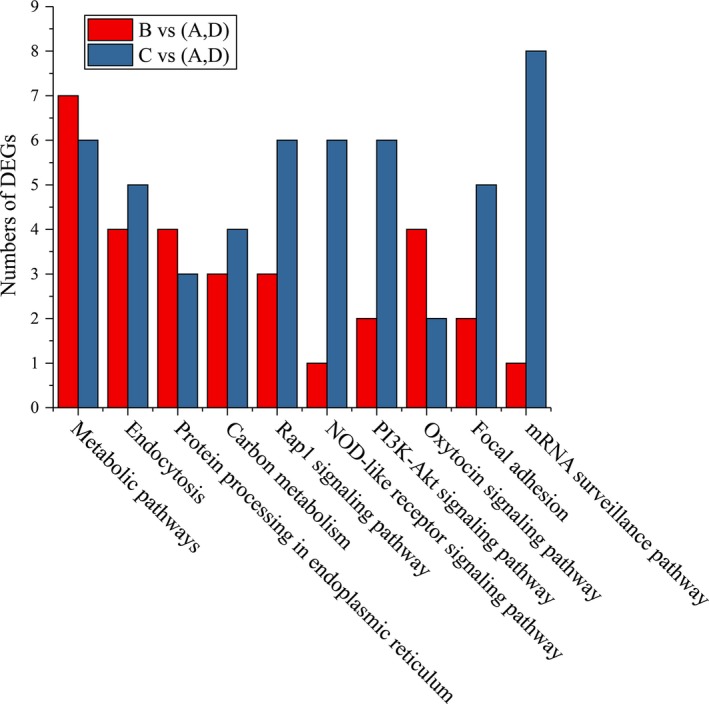
Enriched KEGG pathways of DEGs in the hybrid B‐ and C‐inbred triads.

Differentially expressed genes between the hybrid and inbred families may be involved in heterosis. GO analysis revealed that the DEGs in the B‐inbred triad were involved in the cellular process and metabolic processes in biological processes; in the cell and cell part of the cellular component; and in the binding and catalytic activities in molecular function. The enriched KEGG pathway of the DEGs in the B‐inbred triad indicated that the DEGs were dominantly enriched in the metabolic pathways, including the Focal adhesion and the PI3K‐Akt signaling pathway (Fig. 2). The genes in the enriched KEGG pathway were involved in immune response (nine genes) and growth and development (13 genes).

Gene ontology analysis showed that the DEGs in the C‐inbred triad were involved in the cellular process and metabolic processes in the biological processes; the cell part, organelle, and membrane in the cellular component; and the binding and catalytic activities in molecular function. The enriched KEGG pathway of the DEGs in the C‐inbred triad included the Focal adhesion, PI3K‐Akt signaling pathway, and mRNA surveillance pathway (Fig. 2). The enriched genes in the KEGG pathway were involved in growth and development (nine genes) and immune response (22 genes).

### Validation of differentially expressed genes using qRT‐PCR

**Figure 3:**
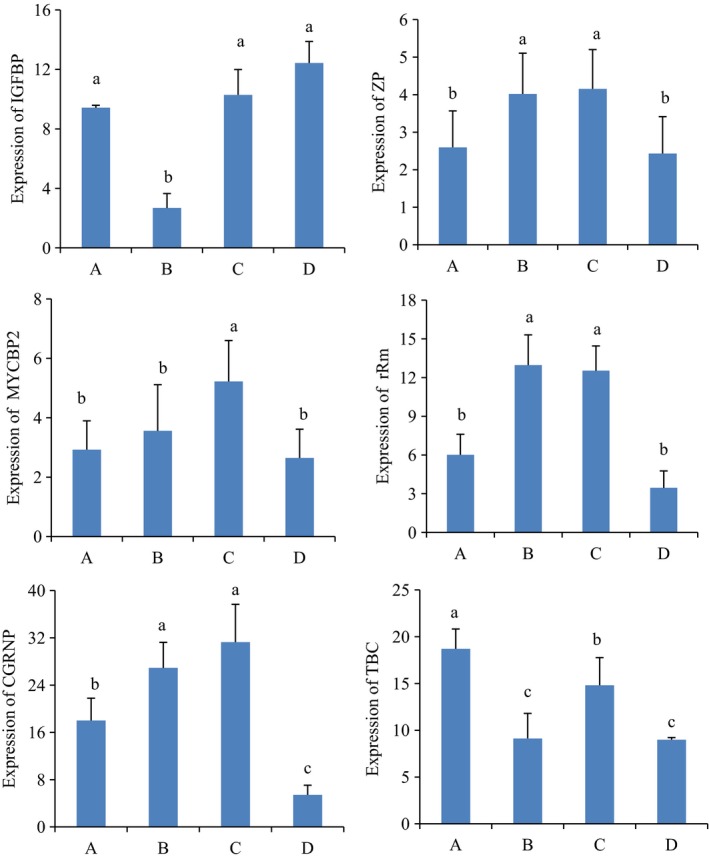
qRT‐PCR validation of the expression levels of six growth‐related genes selected from the DEGs in the B‐ and C‐inbred triads. CGRNP, cell growth‐regulating nucleolar protein; IGFBP, insulin‐like growth factor‐binding protein; MYCBP2, E3 ubiquitin–protein ligase MYCBP2; rRm, ribosomal RNA methyltransferase; TBC, tubulin beta chain; ZP, zinc protein; A and B are inbred families A and D. B and C are hybrid families. Means with the same letter within a column are not significantly different (*P *>* *0.05). Error bars represent SD, *n* = 10.

Quantitative real‐time PCR was utilized to verify the differential expression of genes identified by transcriptome analysis. Six growth‐related genes were selected for qRT‐PCR analysis. These genes included insulin‐like growth factor‐binding protein (*IGFBP*), cell growth‐regulating nucleolar protein (*CGRNP*), E3 ubiquitin–protein ligase MYCBP2 (*MYCBP2*), zinc protein (*ZP*), tubulin beta chain (*TBC*), and ribosomal RNA methyltransferase (*rRm*). The RT‐qPCR expression patterns of the selected DEGs were consistent with the results of RNA‐seq analysis, indicating the reliability and accuracy of the RNA‐seq method used in this study (Fig. 3 and Table 6).

**Table 6:** Differentially expressed candidate genes between the inbred and hybrid families

Sequence ID	Annotation	FPKM			
		A	B	C	D
PINCT_32260	Insulin‐like growth factor‐binding protein	8.07 ± 4.86^a^	1.71 ± 1.18^a^	2.78 ± 1.40^ab^	10.02 ± 1.08^b^
PINCT_23260	E3 ubiquitin–protein ligase MYCBP2	1.26 ± 0.17^a^	1.22 ± 0.07^a^	2.12 ± 1.26^b^	1.27 ± 0.70^a^
PINCT_18317	Cell growth‐regulating nucleolar protein	2.48 ± 0.11^a^	9.03 ± 1.90^b^	6.61 ± 2.24^b^	0.76 ± 0.47^a^
PINCT_08355	Zinc finger CCCH	9.59 ± 0.21^a^	23.06 ± 0.26^b^	21.49 ± 3.17^b^	18.38 ± 5.24^b^
PINCT_32812	ribosomal RNA methyltransferase	15.76 ± 1.41^a^	61.16 ± 1.70^b^	41.65 ± 15.45^b^	30.42 ± 6.23^a^
PINCT_09305	Tubulin beta chain	15.17 ± 2.47^a^	5.45 ± 5.20^b^	8.05 ± 7.36^ab^	0.31 ± 0.15^ab^

Means with the same letter within a column are not significantly different (*P *>* *0.05), Values are means ± SD, *n* = 3.

## Discussion

### MPH values of the inbred and hybrid families

Hybridization between geographically isolated populations, strains, or families is typically used to improve the growth performance and survival of cultured stocks in shellfish species, such as catarina scallop *Argopecten circularis* 18, and bay scallop *Argopecten irradians irradians* 19, Pacific oyster *Crassostrea gigas* 6, 20, Pacific abalone *Haliotis discus hannai* 7, 21, sea scallops *Placopecten magellanicus* 22, and clam *Meretrix meretrix* 8. For example, Deng *et al*. 21 developed a complete diallel cross among three geographically isolated populations (Dalian and Qingdao of China and Miyagi of Japan) of Pacific abalone *H*. *discus hannai*. They reported that the MPH values of shell length, shell width, and total weight are in the ranges of 0.5% to 12.2%, 0.7% to 16.7%, and −1.7% to 44.8%, respectively 21. By crossing three strains of *C. gigas* that were successively mass selected for two generation from three culture stocks collected from China, Japan, and South Korea, Kong *et al*. 20 reported that the MPH values of shell height, shell length, and the whole body weight are in the ranges of −6.07% to 22.35%, −10.46% to 29.74%, and −27.76% to 89.23%, respectively. In the present study, the MPH values (heterosis) of shell length, shell height, shell width, shell weight, and total weight were 12.9%, 14.9%, 18.2%, 19.3%, and 33.2%, respectively. These findings are consistent with those of previous studies on other shellfish species. The MPH values reported here were considerably higher than the results of Wang *et al*. 23. They constructed a 2 × 2 complete diallel cross between Indian and Chinese Sanya wild populations of pearl oyster *Pinctada martensii*. The MPH values of shell height, shell length, shell width, and shell weight were 4.08%, 2.78%, 7.27%, and 6.81%, respectively 23. The present results indicated that the heterosis effects in pearl oyster *P*. *fucata martensii* could be explored by family mating.

### DEGs of the purebred and hybrid families

The genetic basis of heterosis is complex and has yet to be fully elucidated. Several studies have explored the mechanisms underlying heterosis via diallel cross design in shellfish species 4, 6, 8, 21. For example, in a 3 × 3 complete diallel cross by sampling parents from three full‐sib families of the clam *Meretrix meretrix*, Dai *et al*. 8 reported that nonadditive genetic effects are the main causes of genetic variation for shell length and whole body weight. By designing a complete diallel cross between three geographically isolated populations (Dalian and Qingdao of China and Miyagi of Japan) of Pacific abalone *H*. *discus hannai*, Deng *et al*. 21 reported substantial variance in the specific combining ability for growth traits of Pacific abalone *H*. *discus hannai*. Hedgecock and Davis 6 designed four incomplete diallel crosses and factorial mating using a set of six to nine inbred lines as both male and female parents to analyze the yield heterosis in juvenile (seed) and harvest‐sized adult oysters. In these experiments, the variance in yield was partitioned into additive and nonadditive genetic components, and the specific combining ability was found to be significant in all four crosses. These studies imply that nonadditive gene actions determine these growth traits of the shellfish species mentioned above.

With the development of modern biological technologies, recent works have attempted to explain heterosis at the molecular level. The genetic base of hybrid performance can be mainly or partly due to the complementarity of additive and nonadditive genetic effects 24. Several studies have analyzed heterosis‐associated gene expression in crop and animals by comparing the expression patterns of selected genes in inbred lines and hybrids or by performing high‐throughput gene expression analyses via microarray profiling and transcriptome analyses 25, 26, 27, 28. These studies reported that nonadditive gene expression is prevalent between purebred and hybrid families. Hedgecock *et al*. 28 investigated the gene expression patterns underlying the growth heterosis in two partially inbred (inbreeding coefficient = 0.375) and two hybrid larval populations of the Pacific oyster *C. gigas* that were produced by a reciprocal cross between two inbred families. They categorized 70% of genotype‐dependent patterns of gene expression into classical modes of gene action and found nonadditive patterns accounted for 98% of gene expression 28. In the present study, we found that nonadditive gene expression patterns were prevalent between hybrid B or C and its parents. Combined functional analysis of nonadditive genes and expression pattern analysis of genes enriched in GO terms indicated that nonadditive genes participated in biological process regulation, stimulus response, and transcriptional regulation activity. Consequently, the heterosis in the growth traits observed in this study might be induced by the differential expression of the genes in the hybrids.

### Growth‐ and immune‐related genes

Several studies have confirmed that a positive relationship exists between growth traits and pearl traits (pearl thickness and pearl weight) 29, 30. Pearl traits were unexpectedly enhanced by improving the growth traits of pearl oyster stocks. Thus, growth traits are fundamental factors that must be considered in pearl oyster breeding programs. Growth traits are quantitative traits that are determined by multiple genes. The mining of growth‐related genes can provide insights into the underlying mechanisms of growth traits and assist in breeding design. Transcriptome sequencing has been proven efficient for mining growth‐related genes in pearl oyster 10, 11. In this study, we found several growth‐related genes in the DEGs in hybrid B‐ or C‐inbred triads, such as IGFs, GHSR, and zinc finger protein.

The IGFs are a type of multifunctional cell proliferation regulators that play key roles in regulating invertebrate metabolism, growth, and reproduction 31, 32. As IGFs binding proteins, IGFBPs maintain IGF in the circulation, mediate the regulation of IGF independent activities, and regulate the growth and development of bone and other tissues. Zhang *et al*. 33 found that IGFBPs are present in pearl oyster *P. martensii* and are involved in IPL endocrine system regulation by a feeding experiment. GHSR is a G protein‐coupled receptor, the natural ligand of Ghrelin hormones, and is mainly expressed in the hypothalamus and the pituitary gland 34. The GHSR system is an important pathway for regulating growth hormones, which can promote growth and development as well as immune digestion. Shuto *et al*. 35 demonstrated that GHSR regulates growth hormone secretion and food acquisition in the arcuate nucleus. Zinc finger protein is a transcription factor that is divided into nine types, namely, C2H2, C8, C6, C3HC4, C2HC5, CCCH, C2HC, C4HC3, and C4 36, 37, 38. CCCH zinc finger protein is involved in growth regulation and immune function. OMA‐1 and OMA‐2 in nematodes are involved in oocyte maturation and embryonic development 39, 40. In *C. elegans*, xc3 h‐3b plays an important regulatory role in renal differentiation 41. MCP‐induced proteins, which comprise a new family of CCCH zinc finger proteins, regulate macrophage activation, reduce inflammation, and prevent diseases. In this study, we found that several growth‐related genes were present in the DEGs in the hybrid B‐ and C‐inbred triads, indicating that their gene expression contributed to the growth heterosis in pearl oyster *P*. *fucata martensii*.

Innate and adaptive immune responses are well‐recognized host defense mechanisms of plants, fungi, and animals. Invertebrates rely solely on innate immune responses for defense against pathogens in natural environments. Identifying immune‐related genes and their pathway can elucidate the mechanisms against pathogens and develop pathogen‐resistant strains. In this study, we found several immune‐related genes by analyzing DEGs in the hybrid B‐ and C‐inbred triads, such as ubiquitin (Ub) protein ligase E3, histone H2A, and phytanoyl–CoA. Cheng *et al*. 42 identified a novel Ub protein ligase E3 (CgE3Rv1) from *Crassostrea gigas*. They found that CgE3Rv1 is a Ub protein ligase E3 that is involved in the immune response to LPS and in the interaction with cell surface signaling molecules of the neuroendocrine immune system in oysters. Pellino is a highly conserved E3 class Ub ligase that plays a role in the pathogenesis of WSSV and the antiviral mechanism of shrimp *Litopenaeus vannamei* 43. Histones H2A, H2B, H3, and H4 were found in the shrimp *Litopenaeus vannamei* and catfish *Parasilurus asotus*, hematopoietic histone proteins displaying antimicrobial activity and Cathepsin D and metalloproteinases are involved in immune response 44, 45. A yeast two‐hybrid system was used to search for potential proteins of FKBP52. The results indicated that PAHX is an important candidate for studying cell signaling pathways involving FKBP52 in the presence of immunosuppressant drugs 46. The PhyH‐like expression of Xenopus tadpoles was expressed in autoreactive immune cells that were distributed throughout the body during the refractory period 47.

## Conclusion

The transcriptome analysis of hybrid families and their parental families were conducted using the RNA‐seq technology. We found that DEGs in the hybrids and their parents might be associated with growth heterosis. Analyzing the gene action models between the two hybrids and their parents, we proposed overdominance might play important roles on growth heterosis. The transcriptome data from this study will help to understand of molecular mechanism underlying growth heterosis in pearl oyster *P. fucada martensii*.

## Author contributions

YJ and LS performed the experiment and wrote the draft. LJ designed the experiment. ZZ and YD analyzed and interpreted the data. XD revised the manuscript. All authors approved the final manuscript.

## Conflict of interest

The authors declare no conflict of interest.
